# Activating the Fluorescence of a Ni(II) Complex by Energy Transfer

**DOI:** 10.1021/jacs.3c07716

**Published:** 2024-03-21

**Authors:** Tzu-Chao Hung, Yokari Godinez-Loyola, Manuel Steinbrecher, Brian Kiraly, Alexander A. Khajetoorians, Nikos L. Doltsinis, Cristian A. Strassert, Daniel Wegner

**Affiliations:** † Institute for Molecules and Materials, Radboud University, 6500 GL Nijmegen, The Netherlands; ‡ Institute for Experimental and Applied Physics, University of Regensburg, 93040 Regensburg, Germany; § Institut für Anorganische und Analytische Chemie, University of Münster, 48149 Münster, Germany; ∥ Center for Nanotechnology (CeNTech), University of Münster, 48149 Münster, Germany; ⊥ Institut für Festkörpertheorie and Center for Multiscale Theory and Computation, University of Münster, 48149 Münster, Germany; # Cells in Motion Interfaculty Centre (CiMIC) and Center for Soft Nanoscience (SoN), University of Münster, 48149 Münster, Germany

## Abstract

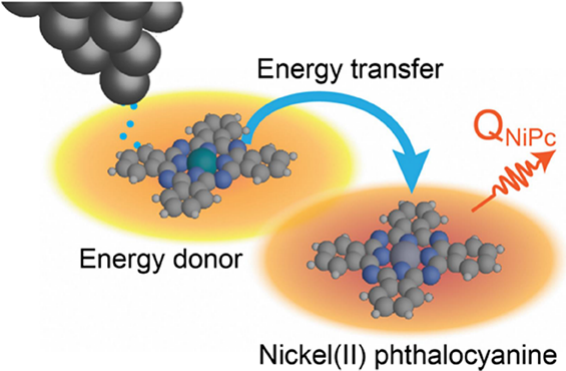

Luminescence of open-shell 3*d* metal complexes is often quenched due to ultrafast intersystem crossing (ISC) and cooling into a dark metal-centered excited state. We demonstrate successful activation of fluorescence from individual nickel phthalocyanine (NiPc) molecules in the junction of a scanning tunneling microscope (STM) by resonant energy transfer from other metal phthalocyanines at low temperature. By combining STM, scanning tunneling spectroscopy, STM-induced luminescence, and photoluminescence experiments as well as time-dependent density functional theory, we provide evidence that there is an activation barrier for the ISC, which, in most experimental conditions, is overcome. We show that this is also the case in an electroluminescent tunnel junction where individual NiPc molecules adsorbed on an ultrathin NaCl decoupling film on a Ag(111) substrate are probed. However, when an MPc (M = Zn, Pd, Pt) molecule is placed close to NiPc by means of STM atomic manipulation, resonant energy transfer can excite NiPc without overcoming the ISC activation barrier, leading to Q-band fluorescence. This work demonstrates that the thermally activated population of dark metal-centered states can be avoided by a designed local environment at low temperatures paired with directed molecular excitation into vibrationally cold electronic states. Thus, we can envisage the use of luminophores based on more abundant transition metal complexes that do not rely on Pt or Ir by restricting vibration-induced ISC.

## Introduction

Molecular luminescence (e.g., fluorescence and phosphorescence) is a ubiquitous phenomenon, providing fundamental insights into light–matter interaction and the electronic and dynamic properties of molecules upon excitation.^1^ Porphyrins and phthalocyanines are a group of chromophores that have historically gained particular attention.^2−4^ In general, the fluorescence from the lowest-ligand-centered singlet (π,π*) excited state (S_1_) competes with radiationless deactivation pathways, including intersystem crossing (ISC) and internal conversion (IC). While IC is impaired for highly rigid closed-shell complexes, ISC is promoted by heavy central atoms and becomes faster as spin–orbit coupling increases. However, the population of dark states that formally involve the occupation of antibonding metal-centered d* orbitals promotes radiationless deactivation pathways by a conical intersection with the ground state. This is a general problem encountered for open-shell 3d transition metal complexes (and somewhat less for 4d elements), with the metallophthalocyanines FePc, CoPc, and NiPc and some of their derivatives being studied particularly well.^2,5−12^ In the case of NiPc, the deactivation of luminescence was explained by the fact that excitation to the S_1_ state is followed by ultrafast (<1 ps) ISC to a vibrationally hot metal-centered (d,d*) state, eventually leading to nonradiative decay to the S_0_ ground state within a lifetime of about 300 ps.^7,8,12^ This efficient conversion of electronic excitation into heat can be used in photothermal therapy and photoacoustic imaging.^13,14^ In optoelectronics, however, luminescent complexes generally rely on expensive and rare elements such as Pt or Ir, providing intrinsically high *d*-orbital splitting paired with high SOC to promote phosphorescence.^15^ Clearly, a sustainable display technology should rely on less critical elements, which is why vast efforts are performed to chemically design complexes accommodating abundant 3d elements,^16,17^ especially Cu(I)^18,19^ and Ni(II).^20−22^ Here, highly rigid luminophoric ligands with optimized ligand-field splittings are used while avoiding the population of dissociative states by pushing the antibonding d* orbitals up in energy.

While the established strategy is to tweak the intramolecular structure chemically toward optoelectronic applicability,^23^ an interesting alternative approach may be the physical design of intermolecular interactions, by controlling the local environment around the luminophore. In this context, recent studies combining scanning tunneling microscopy (STM) with light detection, referred to as STM-induced luminescence (STML) spectroscopy,^24^ not only enabled to fundamentally understand single-molecule fluorescence with submolecular resolution,^25−32^ but especially the influence of the local environment and neighboring chromophores could be investigated using STM-based atomic manipulation techniques. For example, the impacts of adsorption at defects and step edges of an insulating surface on the fluorescence spectrum were studied,^33^ exciton delocalization and superradiance were observed in J-aggregated ZnPc dimers and chains,^25,34,35^ and resonant energy transfer (RET) in molecular donor–acceptor dimers and trimers was investigated with atomic-scale resolution.^36−38^ The latter is particularly interesting, as it may offer the opportunity to activate luminescence in otherwise dark open-shell 3d metal complexes by physical design of the local environment rather than intramolecular chemical design.

In this context, we chose to use NiPc for a proof-of-principle experiment, as the MPc family is well established in STML experiments, permitting stable thermal deposition onto clean substrates in an ultrahigh vacuum environment. We show that the fluorescence of individual NiPc molecules can be activated at low temperatures through resonant energy transfer from neighboring MPc molecules (M = Zn, Pd, Pt). To demonstrate this, we combined STM, atomic manipulation, scanning tunneling spectroscopy (STS), and STML to identify individual NiPc and MPc molecules, to assemble them into NiPc-MPc dimers, and to perform spatially resolved fluorescence spectroscopy. Using three monolayers (ML) NaCl grown on Ag(111) as a substrate, the molecules were sequentially deposited on the surface, and their orbital energies and structure were characterized. We observed no luminescence from individual NiPc molecules, while Q-band emission was detected when NiPc was dimerized with another MPc, and the latter was excited via the STM tunnel current. From spatially dependent STS and STML as well as a comparison of dimers with various intermolecular separations, we determined that RET is responsible for the excitation of NiPc. We explain the emergence of fluorescence by deactivation of the ISC, which is caused by a combination of freezing the relevant molecular vibrations and providing insufficient energy in the RET process to overcome the ISC activation barrier. Thus, the results suggest that a local environment at the cold interface providing precisely tuned energy funneling into the vibrationally “cold” electronic state responsible for the emission of light enables the rather rare emission from an otherwise “dark” 3d metal complex.

## Results

**Figure 1:**
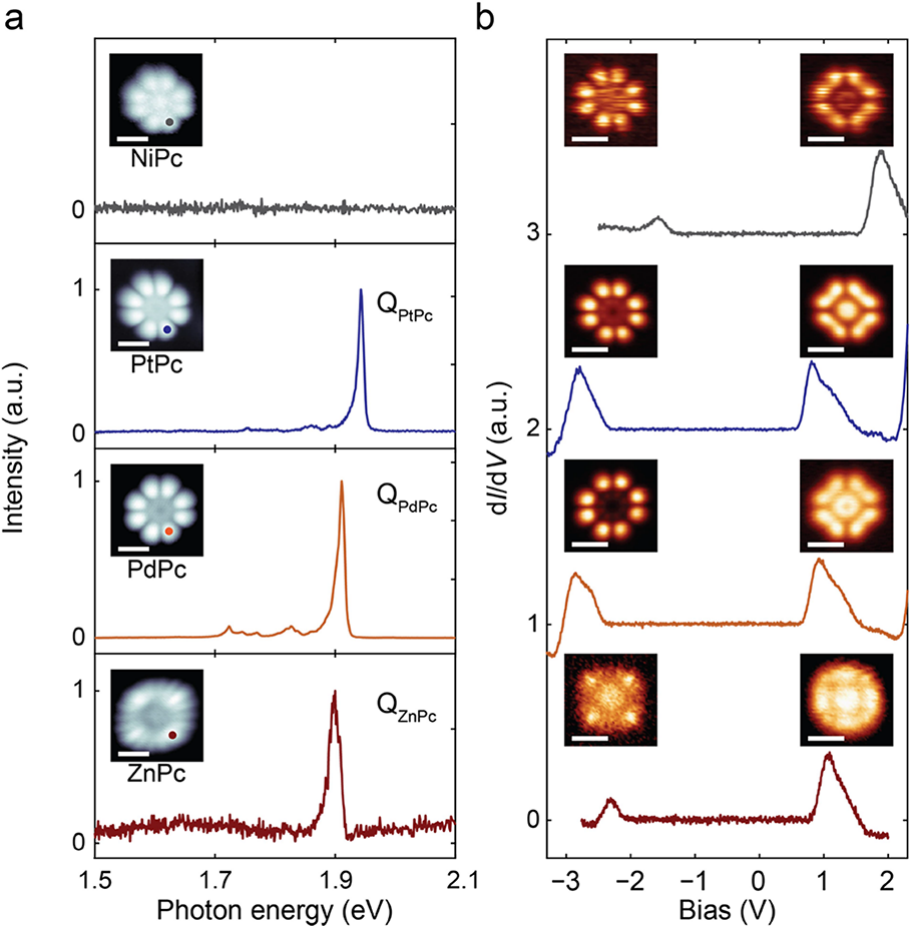
STM-based optical and electronic characterization of individual MPc molecules. (a) STML spectra of NiPc, PtPc, PdPc, and ZnPc on 3 ML NaCl/Ag(111). While NiPc did not show any fluorescence, the other MPc’s exhibited the well-known Q(0,0) fluorescence. The insets show constant-current STM images of the respective MPc molecules, marking positions where the tip was parked for the STML measurements. (b) Differential conductance spectra of the MPc molecules shown in (a), showing the positions of the HOMO and LUMO as found in STS. The insets to the left (right) show the spatial d*I*/d*V* maps of the HOMO (LUMO) orbitals for each molecule, respectively, confirming the assignment. (Parameters used for the data acquisition are given in the Supporting Information, Section 1.)

We first characterized the structural, optical, and electronic properties of NiPc and all MPc monomers by means of STM, STML, and STS at *T* = 4.5 K. Figure 1a shows STM images (inset) as well as STML spectra of individual NiPc, PtPc, PdPc, and ZnPc molecules adsorbed on 3 ML NaCl/Ag(111). The STM topography images taken at a sample bias voltage *V*_s_ = −2.5 V looked similar for all molecules, mostly reflecting the spatial distribution of the highest occupied molecular orbital (HOMO) (see also the spatial maps in Figure 1b). Note that the image of ZnPc reflects the superposition of two different adsorption orientations, as the molecule rapidly shuttles between them;^33^ contrastingly, NiPc, PtPc, and PdPc did not show rapid shuttling, and the aromatic rings are oriented along the NaCl ⟨110⟩ crystallographic directions. However, one peculiar difference seen for NiPc was an increased noise level in the constant-current topography. This was not seen on the other MPc molecules, even when measured with the same tip and identical imaging parameters (Figure 3). As this apparent instability was reproduced using more than a dozen different microtips, this observation cannot be a tip artifact but is intrinsic to NiPc. We note that the noisy appearance was observed for all voltages, and a histogram analysis did not show discrete telegraph noise-like steps that would be indicative of switching events.

STML spectra reproduced the single-molecule fluorescence observed in previous studies of ZnPc,^25,29,33,34^ PdPc,^36^ and PtPc^38^ adsorbed on ultrathin NaCl films, illustrating that the tip used is properly calibrated for STML (Figure 1a). The most prominent spectral feature is the <20 meV-sharp Q(0,0) resonance emitted upon relaxation of the given MPc from the S_1_ excited singlet state to the S_0_ ground state, with a maximum at *E*(Q_PtPc_) = 1.94 eV, *E*(Q_PdPc_) = 1.91 eV, and *E*(Q_ZnPc_) = 1.90 eV, for each of the respective molecules. In contrast, NiPc did not show any Q-band emission (top spectrum in Figure 1a). To verify this, we also checked STML spectra at other sample bias voltages *V*_s_, including at positive polarity (see the Supporting Information, Section 2), but we could not find any emission for NiPc in all studied parameter space.

The different character of NiPc compared to the other MPc molecules is also reflected in STS. As shown in Figure 1b, d*I*/d*V* spectra of the other MPc molecules looked similar, with one peak visible below the Fermi level (*E*_F_) at *V*_s_ < – 2 V and a second peak found above (*E*_F_) at *V*_s_ ≈ 1 V. These peaks are the positive (PIR) and negative ion resonances (NIR) when tunneling out of the HOMO or into the lowest unoccupied molecular orbital (LUMO), respectively. This was further confirmed by d*I*/d*V* maps taken at the respective peak voltages, reflecting the spatial distributions of HOMO and LUMO (see the inset images in Figure 1b). The electronic HOMO–LUMO gap (determined by identifying the onset energies^27^) varied from 2.85 eV (ZnPc) to 3.0 eV (PtPc) and to 3.05 eV (PdPc). NiPc also displayed two peaks in STS, but at very different voltages. The onset of the PIR was found at *V*_s_ = −1.30 V (maximum at −1.6 V), significantly closer to *E*_F_ than that for the other MPc molecules. The NIR onset was located at *V*_s_ = 1.55 V (maximum at 1.9 V). Hence, the electronic HOMO–LUMO gap of NiPc was identical to that of ZnPc, but the peaks were shifted higher in energy by about 1 eV. We will discuss the consequences of this for STML further below. We discuss a possible explanation for the relatively large shift of the NiPc d*I*/d*V* spectrum in the Supporting Information (Section 2).

**Figure 2:**
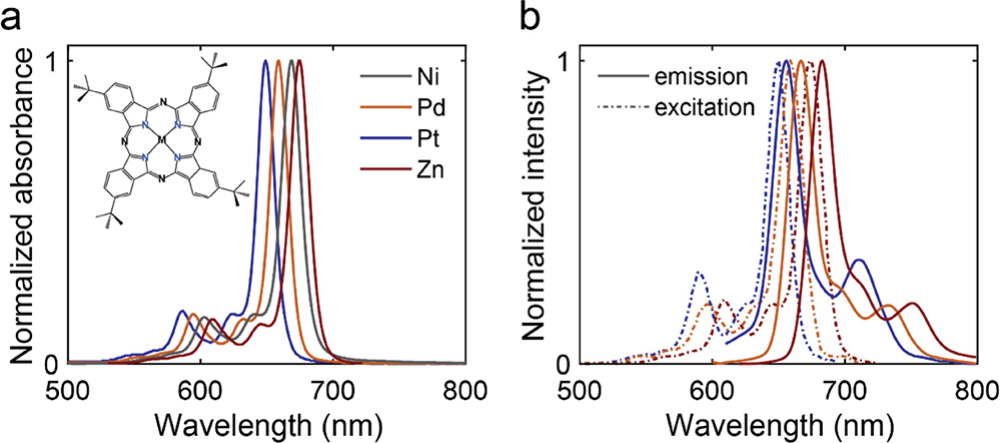
Optical properties of phthalocyanines in solution. (a) Absorption spectra of NiPc-(*t*Bu)_4_ and MPc-(*t*Bu)_4_ (M = Zn, Pd, and Pt) in a 2Me-THF:toluene solution taken at room temperature. The *tert*-butyl groups have been added for solubility reasons but should not significantly affect the optical properties. (b) Fluorescence emission (solid line) and excitation (dashed line) spectra of MPc-(*t*Bu)_4_ at room temperature. For NiPc-(*t*Bu)_4_, no emission was found.

In order to confirm that the lack of fluorescence from NiPc is not related to the STML technique, we performed separate optical absorption and photoluminescence spectroscopy of monomer ensembles of the derivative NiPc-(*t*Bu)_4_ and MPc-(*t*Bu)_4_ in solution at room temperature (Figure 2). We note that the *tert*-butyl groups were necessary to improve the solubility of the molecules, but as they are located at β-positions of the Pc macrocycle (see the inset in Figure 2a for the molecular structure), they should not significantly affect the optical properties.^39^ From the absorption spectra (Figure 2a), the Q-band of NiPc-(*t*Bu)_4_ is located at an energy similar to that of the other MPc molecules. However, fluorescence emission and excitation spectroscopy (Figure 2b) did not reveal any Q-band emission for NiPc-(*t*Bu)_4_. As the STML experiments were performed at low temperatures, we also performed optical spectroscopy in solution at 77 K (see Figure S3) and in PMMA matrices down to *T =* 6 K. The absence of luminescence in all cases confirms that NiPc-(*t*Bu)_4_ is nonfluorescent, even when optically excited from a vibrational shoulder only 0.28 eV higher in energy than the NiPc-(*t*Bu)_4_ Q-band.

**Figure 3:**
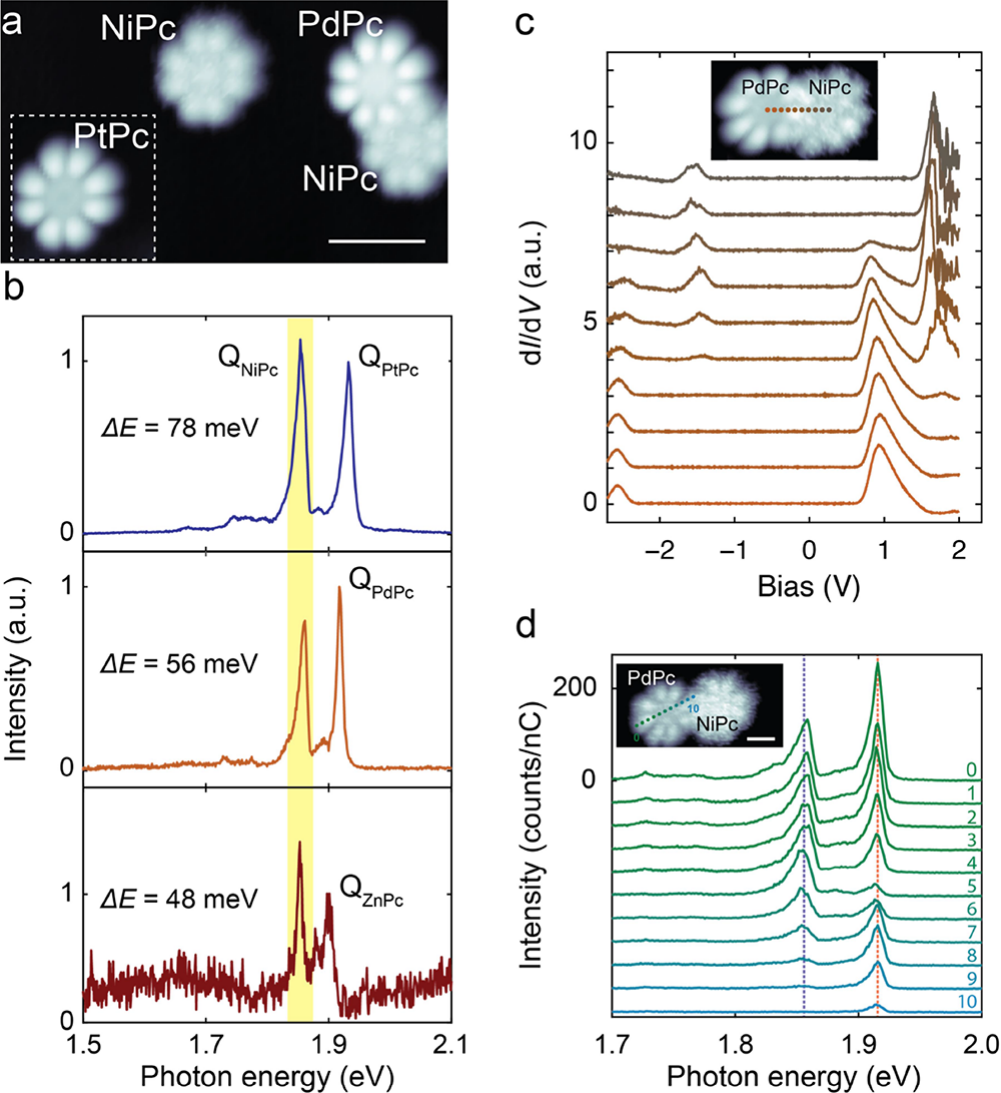
Resonant energy transfer and fluorescence of NiPc in MPc dimers. (a) Constant-current STM image showing MPc monomers and a PdPc-NiPc dimer with center-to-center distance *R* ≈ 1.43 nm (scalebar: 2 nm; stabilization parameters: *V*_s_ = −2.5 V, *I*_t_ = 10 pA). (b) STML spectrum of the MPc-NiPc dimer with M = Pt (top), Pd (center), and Zn (bottom), respectively. The tip was always parked above the MPc. In addition to the Q-band emission of the respective MPc, a second sharp emission peak Q_NiPc_ was observed, highlighted by the yellow background (*I*_t_ = 100 pA, *V*_s_ = −2.6 V, *t* = 120 s). (c) Set of d*I*/d*V* spectra taken across a PdPc-NiPc dimer (see the inset), revealing that the individual molecular orbitals are not altered compared to isolated monomers (feedback loop opened at the center of PdPc with *V*_s_ = −2.8 V, *I*_t_ = 50 pA). (d) Set of STML spectra taken across a PdPc-NiPc dimer with *R* ≈ 1.85 nm. Q_NiPc_ emission was strongest when the tip was parked on the PdPc with maximum distance to the NiPc, and it vanished as soon as tunneling into NiPc was possible (*I*_t_ = 100 pA, *V*_s_ = −2.5 V, and *t* = 120 s).

Next, we show that the fluorescence of NiPc can be activated by energy transfer. We built dimers of NiPc with the other MPc molecules, as shown in Figure 3a, with typical distances (center to center) between the molecules of about *R* = 1.45 ± 0.09 nm. When the tip was positioned above NiPc within the dimer, there was still no detectable luminescence. However, when the tip was parked on the lobe of the adjacent MPc molecule, two distinct resonance peaks appeared in the STML spectra (Figure 3b). In all cases, the high-energy peak was identical to the Q(0,0) resonance of the respective MPc monomer, while the low-energy peak was always located at a photon energy of 1.86 eV. As this low-energy peak only appeared in the presence of the NiPc molecule and is independent of the chosen MPc molecule, this is a clear sign that this resonance originates from the NiPc molecule and corresponds to its Q-band emission, i.e., *E*(Q_NiPc_) = 1.86 eV. NiPc was excited into its S_1_ state via a RET process from the MPc and then radiatively decayed to the ground state. In this scenario, the individual MPc molecules serve as donors exhibiting a higher exciton energy, and the NiPc is the acceptor, i.e., an exciton can be transferred from the MPc to NiPc, but not the other way around.^36−38^

To verify that the dimers were not electronically hybridized, namely, strongly bonding, we performed a series of STS spectra along a line across the dimer. Figure 3c shows an example of a PdPc-NiPc dimer (see Figure S5 for a corresponding data set on a ZnPc-NiPc dimer). We found that in all cases the PIR and NIR positions were identical to those of the monomers, as shown in Figure 1. In the region where the two molecules are closest to each other, no continuous transition of the PIR and NIR positions occurred, but the d*I*/d*V* spectra showed PIR and NIR from both molecules. This coexistence results from a finite tunneling probability into either of the two molecules, when the tip is located in between the two. This is also confirmed in the STM images, where it can be seen that there is a spatial overlap of the orbital features in this region. Hence, the individual molecular orbital structures were not altered in the dimer, compared to isolated monomers, and therefore, the resultant peaks in the STML spectra do not result from a hybrid electronic structure. To further confirm that the NiPc emission is due to a RET mechanism, we also acquired STML spectra as a function of intermolecular distance between a ZnPc and a NiPc molecule (see the Supporting Information, Section 4 and Figure S4) and found that the distance-dependent RET efficiency is similar to that of previous studies.^36,38^

The RET efficiency varies with the position of the tip with respect to the MPc donor molecule.^36,38^ In Figure 3d, we present STML spectra and extracted Q-band intensities taken at different positions of a PdPc-NiPc dimer. Both the Q_PdPc_ and Q_NiPc_ emissions were strongest when the tip was placed on PdPc, with a maximum distance to the NiPc. As the tip moved closer to the NiPc, the Q_NiPc_ emission initially remained constant up until the center of PdPc was reached, and then it continuously decreased and eventually vanished as soon as direct tunneling from NiPc was probable. Compared to that, the Q_PdPc_ emission continuously decreased from the edge of the molecule farthest from NiPc until the PdPc center was reached. From there, the intensity recovered slowly but eventually also tended to vanish when tunneling from NiPc to the tip became dominant.

We note that also homodimers of two NiPc molecules showed no fluorescence in STML (see Figure S6a). In comparison, homodimers of ZnPc^32^ and PdPc (Figure S6b) showed a redshift and sharpening of the Q-band emission, which is an expected effect of coherent excitonic coupling.^25,34^ Overall, our observations are in line with previous submolecularly resolved STML studies of Pc-based donor–acceptor dimers, confirming our interpretation that the fluorescence of NiPc is enabled by RET from one of the MPc donor molecules (M = Zn, Pd, or Pt).

## Discussion

**Figure 4:**
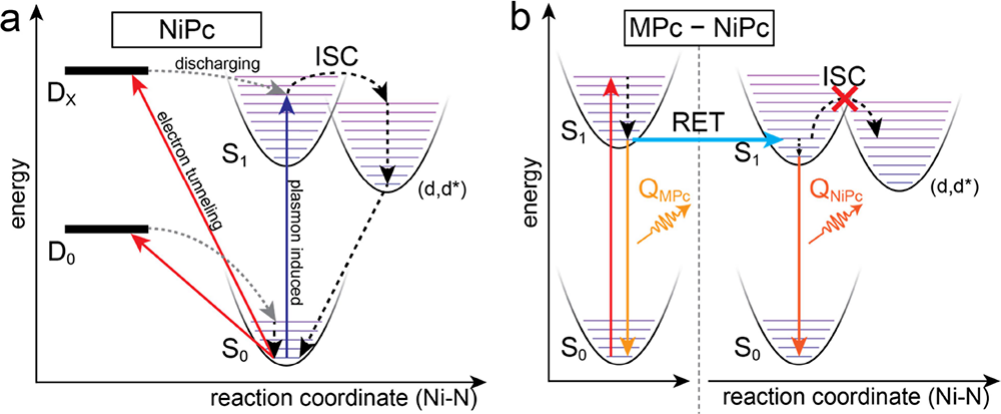
Energy diagram of NiPc excitations and energy transfer. (a) Schematic potential energy diagram of the NiPc monomer showing tunneling- (red arrows) or plasmon-induced (blue) excitation channels that lead to ISC and radiationless deactivation. (b) Schematic potential energy diagram of the MPc-NiPc dimer showing how RET activates radiative decay from the S_1_ state by preventing ISC.

The activation of NiPc fluorescence via RET can be understood by reviewing the many-body energy diagram of NiPc and considering the various pathways that lead to its excitation. Figure 4a shows the potential energy of the most relevant NiPc states as a function of the reaction coordinate, which is mostly the Ni–N_p_ bond distance between the central Ni atom at its four neighboring isoindole N atoms in the center of the Pc macrocycle (see below).^12^ Our observations can be rationalized by assuming a small energy barrier between the vibrationally cooled S_1_ and the (d,d*) state, which has to be overcome to activate the ISC, e.g., by exciting molecular vibrations. In any photoluminescence experiment, an excitation photon energy must be chosen that is larger than the fluorescence energies. Hence, there is sufficient excess energy to overcome this activation barrier. Thus, depending on the energy of the absorbed photon, a vibrationally excited state within the S_1_ electronically excited state is reached either directly or via excitation into a higher electronically excited state (e.g., S_2_) followed by relaxation into a vibrationally hot S_1_ state.^8,10,11^ The ultrafast ISC into the (d,d*) state is possible when the vibrationally excited S_1_ level is above the activation barrier, leading to radiationless deactivation. This scenario can also explain why optical spectroscopy experiments even at low temperatures still lead to the absence of NiPc fluorescence (see the Supporting Information, Section 3).

In STML experiments of isolated NiPc molecules, the excitation is mostly preceded by an electron tunneling into (or out of) the molecule, leading to an anionic (or cationic) doublet state, which is drawn schematically in Figure 4a (red arrows).^27,32^ In cases where the doublet ground state D_0_ has a lower energy than the S_1_ state, the molecule can only go back to S_0_ by a nonradiative discharging process.^32^ In NiPc, both the NIR and PIR threshold magnitudes are accessed at voltages where *eV*_s_ < *E*(Q_NiPc_); hence, both anionic and cationic D_0_ levels are lower than S_1_. A similar situation was previously found for other nonluminescent Pc molecules.^40,41^ The S_1_ state may still be accessible by applying larger voltages that allow tunneling out of lower-lying occupied (or into higher-lying unoccupied) orbitals. This excites the molecule into a higher doublet state (D_*x*_) and permits access to S_1_ upon discharging.^32^ We have confirmed this by d*I*/d*V* and STML spectroscopy down to voltages of *V*_s_ = −3 V (see Figure S1), and there is still no detectable NiPc emission despite fulfilling *E*(D_*x*_) > *E*(S_1_) (see also Section 2 of the Supporting Information). However, there is usually an energy mismatch, and therefore, the discharging will end in a vibrationally hot S_1_ state. In the case of NiPc, this again activates ultrafast ISC to the dark (d,d*) state.

Another known excitation channel in STML is plasmon-induced excitation. Molecules can be excited remotely with the tip displaced laterally by a few nanometers. This way, no direct tunneling through NiPc occurs, but the nanocavity plasmons (NCP) can still couple to and excite the molecule to S_1_. We conducted such an experiment, but we did not observe any plasmon-induced luminescence for NiPc (see Figure S7). Obviously, plasmon-exciton coupling can also excite the molecule into a vibrationally hot S_1_ state (blue arrow in Figure 4a), hence overcoming the ISC activation barrier and deactivating NiPc emission.

To activate fluorescence in NiPc, it is important to provide an excitation channel into an S_1_ level that lies below the activation barrier for the ISC. This is possible via RET, as shown schematically in Figure 4b. In a MPc-NiPc dimer, when the tip is positioned above the MPc, the initial excitation via a transiently charged state only occurs in the MPc, as no direct electron tunneling via the NiPc happens. This also results in a vibrationally hot S_1_ state. However, for the MPc molecules used here (M = Zn, Pd, or Pt), there is no lower-lying (d,d*) state. Therefore, the molecule cools into the lowest vibrational S_1_ level, following Kasha’s rule.^34^ From there, either radiative decay into the MPc S_0_ state or an RET process to neighboring NiPc occurs. As the Q(0,0) energies of all MPc molecules are very close to that of NiPc, the RET leads to a NiPc S_1_ level that is below the ISC activation barrier. Importantly, the direct optical (or RET) excitation of (d-d*) states is parity forbidden (Laporte’s rule).

To rationalize the conceptual potential energy diagram used in Figure 4, we performed time-dependent density functional theory (TDDFT) calculations (see the Supporting Information, Section 7). A comparison of the optimized molecular structure in the S_1_ state as well as various possible (d,d*) states revealed larger Ni–N_p_ bond distances in the case of the latter. Hence, we can identify the “reaction coordinate” axis, which was not defined in previous theory work,^12^ as the Ni–N_p_ bond length, as shown in Figure 4. A calculation of the (π,π*) as well as various singlet and triplet (d,d*) excited-state energies as a function of Ni–N distance (Figures S8 and S9) also confirms the three main features of our schematic potential diagram in Figure 4b: (1) the equilibrium Ni–N distance in the (d,d*) states is larger than in the (π,π*) state; (2) almost all of the calculated (d,d*) states in equilibrium are at lower energy than the (π,π*) state; (3) (d,d*) potential curves intersect with the (π,π*) potential, rationalizing an activation barrier that is roughly given by the energy difference between the intersection and the minimum of the (π,π*) state. Unfortunately, TDDFT does not allow us to reliably quantify the activation barrier with the degree of accuracy required here. While the above three features are qualitatively robust in all calculations, the quantitative values of potential minima and intersection points are very sensitive to the functional used. Besides, the gas-phase calculations did not include the influence of the surface, which we expect to slightly steepen the potential curves due to the impact on the Ni–N breathing mode.

Finally, we discuss the magnitude of the ISC activation barrier. Our STML experiments showed the largest difference to the Q_NiPc_ emission energy for Q_PtPc_ with Δ*E* = 78 meV (Figure 3b). As RET was still observed, this defines a lower boundary of the activation barrier, Δ*E*_ISC_ ≥ 0.08 eV. We note that absorption resonances are usually at slightly higher energy than emission resonances, known as Stokes shift (see Figure 2 for Stokes shifts of MPc molecules in solution at room temperature). However, measurements of ZnPc in a cryogenic matrix revealed no detectable Stokes shift,^42^ which is why the impact can likely be neglected here. Looking at the results from our calculations, changing the Ni–N bond distance requires a vibrational excitation of about 0.18 eV corresponding to the frequency of 1410.8 cm^–1^ of the Ni–N breathing mode. The intersection point of the potentials is obviously within the uncertainty range of TDDFT, which is typically about 0.3 eV.^43,44^ We therefore assume this value to be an upper boundary for the ISC activation barrier. This can be confirmed by comparing the smallest photon energy used in our photoluminescence experiments (see the Supporting Information, Section 3) with the NiPc Q-band energy. From this, we can assume an upper boundary of the ISC activation barrier of Δ*E*_ISC_ ≤ 0.28 eV. These estimates indicate that the excitation energy must be tuned within a relatively narrow range to enable NiPc fluorescence.

A further quantification of the ISC activation barrier would require the use of donor molecules with even larger mismatch of the Q-bands, to see at which point it becomes large enough to induce ISC. Another possibility might be an experiment using a carefully tuned tip for remote plasmon-induced excitation. If voltages are applied that excite plasmons with energies *E* < *E*(Q_NiPc_) + Δ*E*_ISC_, then plasmon-induced molecular luminescence might be activated. However, this quantum cutoff energy also dramatically cuts off the NCP intensity at *E*(*Q*_NiPc_), and finding experimental evidence for NiPc fluorescence under such conditions may prove to be extremely difficult.

## Conclusions

In summary, we showed that fluorescence from NiPc molecules can be activated at low temperatures via resonant energy transfer from neighboring MPc molecules. We rationalize this observation by an activation barrier on the order of 0.1 eV for the rapid intersystem crossing from the normally emissive S_1_ to the dark excited state. In most experimental situations, this barrier is readily overcome by vibrational excitations of the molecule, influencing the Ni–N_p_ bond distance. However, if the S_1_ energy of neighboring MPc molecules is only slightly above that of NiPc, then RET can occur without overcoming the ISC activation barrier. This enables radiative decay from S_1_ to the S_0_ ground state and hence Q-band emission of NiPc. This strategy should also work for other compounds that possess rapid deactivation of luminescence, e.g., other open-shell dark metallophthalocyanines such as VPc, FePc, and CoPc, or corresponding metal porphyrins. Beyond that, more systematic studies of the conditions under which RET enables molecular luminescence may shed more light on the underlying mechanisms of intermolecular energy transfer, with relevance in photosynthesis and photovoltaics.^45,46^

Our findings encourage the exploration of rigidified local environments at low temperatures, which permit the directed excitation of the vibrationally “cold” electronic excited states to enable the rather rare emission from dark 3*d* metal complexes. In this context, it would be interesting to develop ultrafast transient absorption spectroscopy toward low-temperature studies of molecules in frozen matrices or on solid-state substrates. This may allow one to further detail and quantify the NiPc excited states energy diagram.^12^ Furthermore, a deeper understanding of the nature and role of molecular energy transfer in electroluminescence enables one to establish intermolecular design strategies toward efficient lighting applications, in addition to the already established strategy to tweak the intramolecular structure.^23^ Thus, the use of rare metals such as Ir or Pt could be replaced by more abundant exemplars involving Ni(II) complexes.^5^

## Supporting Information Available

Experimental details, d*I*/d*V* and bias dependence of STML spectra, temperature-dependent photoluminescence spectra of NiPc, distance dependence of energy transfer between ZnPc and NiPc, STS on ZnPc-NiPc dimers, STML on homodimers, spatial dependence of STML, theoretical details and analysis, and NiPc vs HPc^–^ comparison (PDF)
