# Supplementary material for: Activating the Fluorescence of a Ni(II) Complex by Energy Transfer
Source: J Am Chem Soc. 2024 Mar 21;146(13):8858–64. doi: 10.1021/jacs.3c07716 (PMC10996004; doi:10.1021/jacs.3c07716)
Supplement: Supplementary file 1 — ja3c07716_si_001.pdf [file ja3c07716_si_001.pdf]

Supporting Information

Activating the fluorescence of a Ni(II) complex by energy transfer

Tzu-Chao Hung[1,2], Yokari Godinez-Loyola[3,4], Manuel Steinbrecher[1], Brian Kiraly[1], Alexander A. Khajetoorians[1], Nikos L. Doltsinis[5], Cristian A. Strassert[3,4,6], and Daniel Wegner[1*]

Affiliations:

[1] Institute for Molecules and Materials, Radboud University, 6500 GL Nijmegen, The Netherlands

[2] Institute for Experimental and Applied Physics, University of Regensburg, 93040 Regensburg, Germany

[3] Institut für Anorganische und Analytische Chemie, University of Münster, 48149 Münster, Germany

[4] Center for Nanotechnology (CeNTech), University of Münster, 48149 Münster, Germany

[5] Institut für Festkörpertheorie and Center for Multiscale Theory and Computation, University of Münster, 48149 Münster, Germany

[6] Cells in Motion Interfaculty Centre (CiMIC) and Center for Soft Nanoscience (SoN), University of Münster, 48149 Münster, Germany

[*] Email: d.wegner@science.ru.nl

The supporting information includes:

1. **Experimental details**
2. **d*I*/d*V* spectra and bias dependence of STML**
3. **Temperature-dependent photoluminescence spectra of NiPc**
4. **Distance dependence of energy transfer between ZnPc and NiPc**
5. **STS on ZnPc-NiPc dimers**
6. **STML on homodimers**
7. **Spatial dependence of STML**
8. **Theoretical details and analysis**
9. **NiPc vs. HPc^{-}**

1. Experimental details

ZnPc was purchased from Sigma-Aldrich. NiPc PdPc and PtPc as well as NiPc-(*t*Bu)$_4$, ZnPc-(*t*Bu)$_4$, PdPc-(*t*Bu)$_4$ and PtPc-(*t*Bu)$_4$ were kindly supplied by PorphyChem (Dijon, France).

The STM experiments were carried out in a commercial Omicron ultra-high vacuum (UHV) low-temperature STM system operated at $T = 4.5$ K with a base pressure below 1×10^{-10} mbar.[1] We used a silver bulk tip, which was electrochemically etched in a mixture of perchloric acid and methanol (ratio 1:4),[2,3] and further treated in UHV by field emission and controlled indentation on a Ag(111) surface. The Ag(111) single crystal (MaTeck) was cleaned by multiple cycles of sputtering and annealing followed by NaCl deposition while the Ag(111) surface was kept at room temperature. The NiPc and MPc molecules were successively sublimated from a Knudsen cell evaporator and deposited on the surface held at $T < 6$ K inside the STM. We note that the evaporator was always thoroughly degassed in UHV by heating to the target temperature for tens of minutes (in a UHV space decoupled from the main UHV chambers), in order to reduce contaminations in the source (see also Section 9).

All STML spectra were acquired using a 150 grooves/mm grating. Further details of the optical setup can be found in a previous publication.[1] PdPc-NiPc and ZnPc-NiPc dimers were created by lateral atomic manipulation of the PdPc and ZnPc molecules, respectively, following recipes reported in the literature.[4,5] We note that we were not able to reliably manipulate NiPc molecules. The PtPc-NiPc dimer whose STML spectrum is shown in Fig. 2 was found self-assembled on the surface.

The STML spectra presented in Fig. 1a were acquired in constant-current mode using the following stabilization voltage (V_s), tunnel current (I_t) and acquisition time t: $V_s = -2.5$ V, $I_t = 100$ pA, $t = 300$ s (NiPc); $V_s = -2.6$ V, $I_t = 50$ pA, $t = 120$ s (PtPc); $V_s = -2.5$ V, $I_t = 100$ pA, $t = 120$ s (PdPc and ZnPc). The inset constant-current topography images were acquired using $V_s = -2.6$ V, $I_t = 10$ pA (NiPc and PtPc) and $V_s = -2.5$ V, $I_t = 10$ pA (PdPc and ZnPc).

The dI/dV spectra shown in Fig. 1b were acquired with the feedback loop opened at the center of the molecule using: $V_s = +2.5$ V, $I_t = 200$ pA (NiPc); $V_s = -3.5$ V, $I_t = 100$ pA (PtPc); $V_s = -3.0$ V, $I_t = 100$ pA (PdPc); $V_s = -2.8$ V, $I_t = 50$ pA (ZnPc). The inset constant-height dI/dV maps were taken with the tip stabilized above the center of each molecule prior to opening the feedback loop, at $I_t = 100$ pA and V_s set close to the respective maximum of the HOMO/LUMO peak, which was –1.5 V/+1.5 V (NiPc), –2.5 V/+0.7 V (PtPc), –2.4 V/+0.85 V (PdPc), and –2.25 V/+0.8 V (ZnPc), respectively.

For the photophysical characterization, all solvents used were of spectroscopic grade (Uvasol®). Absorption spectra were measured with a Shimadzu UV-3600 I plus UV-VIS-NIR spectrophotometer. Steady-state excitation and emission spectra were recorded on a FluoTime300 spectrometer from PicoQuant equipped with a 300 W ozone-free Xe lamp (250-900 nm), a 10 W Xe flash-lamp (250-900 nm, pulse width ca. 1 µs) with repetition rates of 0.1 – 300 Hz, a double-grating excitation monochromator (Czerny-Turner type, grating with 1200 g/mm, blaze wavelength: 300 nm), two double-grating emission monochromators (Czerny-Turner, selectable gratings blazed at 500 nm with 2.7 nm/mm dispersion and 1200 grooves/mm, or blazed at 1200 nm with 5.4 nm/mm dispersion and 600 grooves/mm) with adjustable slit width between 25 µm and 7 mm, Glan-Thompson polarizers for excitation (Xe-lamps) and emission (after the sample). Different sample holders (Peltier-cooled four-position cuvette sample holder ranging from -15 to 110 °C and round cuvette sample holder), along with two

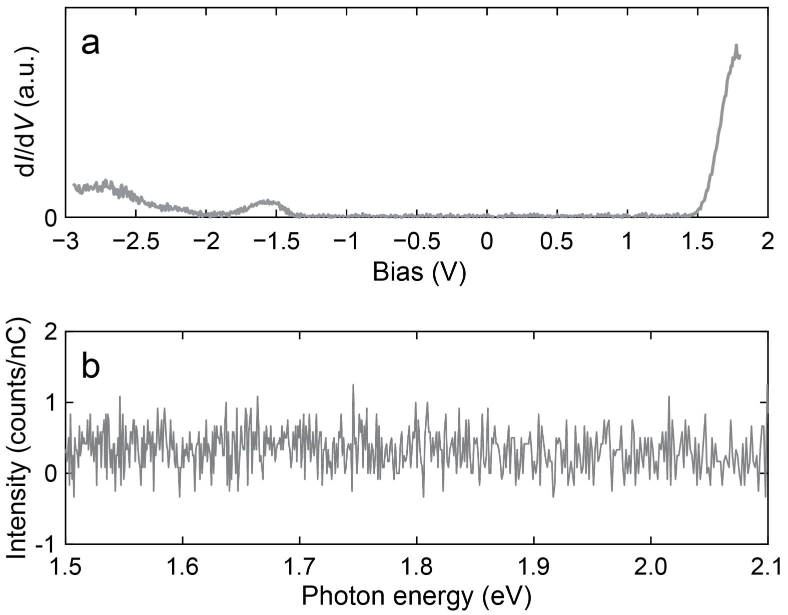

Figure S1. Large negative bias range STS and STML spectra. (a) d*I*/d*V* spectrum on the center of NiPc adsorbed on 3 ML NaCl/Ag(111), extended down to –3 V (feedback loop opened at NiPc center with $V_s = -2.6$ V, $I_t = 100$ pA). An additional peak can be found at –2.7 V, and a shoulder is visible at about –2.3 V. (b) STML spectrum acquired on NiPc on 3 ML NaCl/Ag(111) at large negative sample bias ($V_s = -3$ V, $I_t = 100$ pA, $t = 120$ s).

detectors, namely a PMA Hybrid-07 (transit time spread FWHM < 50 ps, 200 – 850 nm) and an H10330C-45-C3 NIR detector (transit time spread FWHM 0.4 ns, 950-1700 nm) from Hamamatsu were used. Emission and excitation spectra were corrected for source intensity (lamp and grating) by standard correction curves.

2. d*I*/d*V* spectra and bias dependence of STML

An obvious difference of NiPc over the other MPc molecules is a relatively large shift of the PIR and NIR by about 1 V, and we would like to provide a hypothesis for this. In the energy range of the NiPc frontier orbitals, several d orbitals can be found from theory (see also Section 8 and Table S2).[6] Especially the d_π orbitals (i.e. d_{xz} and d_{yz}) can have a relatively strong interaction with the ligand-π orbitals, which could shift the positions of the latter. PdPc and PtPc have a much larger ligand field splitting, owing to the 4d and 5d character (as opposed to 3d for NiPc). Therefore, their d_π orbitals are lying deeper in energy and do not influence the frontier orbitals so much. As ZnPc has a completely filled d-shell, its d orbitals are also at lower energies. To confirm this hypothesis, d*I*/d*V* spectra of other 3d transition metal phthalocyanines, e.g. MnPc, FePc and CoPc should be compared with each other on NaCl/Ag(111). In this context, we note that a comparison of MnPc with MgPc molecules on NaCl/Cu(111) showed a comparably strong shift in the d*I*/d*V* spectra.[7]

Fig. S1a shows a d*I*/d*V* spectrum acquired on NiPc extended down to −3 V, which indicates the presence of at least two occupied molecular orbitals below −2 V. Hence, tunneling an electron out of one of those orbitals excites the NiPc molecule into a transiently charged (i.e. $NiPc^{+}$) doublet state D_x that fulfills the condition $E(D_x) > E(S_1)$. This shows that already the STML spectra taken at −2.5 V (Fig. 1a) and −2.6 V (Fig. S4 and S5) provide evidence that NiPc

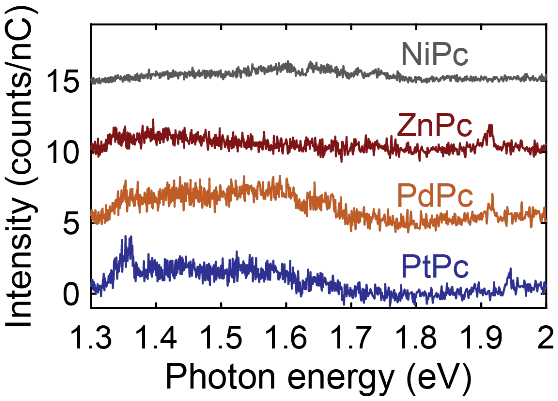

Figure S2. Bias dependence of STML spectra. STML spectra acquired on 3 ML NaCl/Ag(111) at positive sample bias of NiPc (grey, $V_s = 2.5$ V, $I_t = 100$ pA, $t = 300$ s.), ZnPc (red, $V_s = 2.5$ V, $I_t = 50$ pA, $t = 300$ s.), PdPc (orange, $V_s = 2.6$ V, $I_t = 100$ pA, $t = 120$ s.), and PtPc (blue, $V_s = 2.5$ V, $I_t = 100$ pA, $t = 120$ s.). The spectra are offset for clarity.

in non-emissive in STML. To confirm this, we provide STML spectra taken at −3 V (Fig. S1b). Again, the NiPc is non-emissive even under such extreme condition.

To verify that individual NiPc molecules on 3 ML NaCl/Ag(111) do not show any Q-band emission, irrespective of the tunneling conditions, we also acquired STML spectra at positive sample bias. As shown in Fig. S2, no STML intensity was found around $E(Q_{NiPc})$ = 1.86 eV. The spectrum only contained weak and spectrally broad features stemming from radiative NCP decay. We note, however, that at positive bias it is not clear if a magnitude of 2.5 V is sufficient to access an orbital that excites NiPc into a doublet state that is higher in energy than the S_1 state. If we were to assume a rigid shift of all ligand orbitals, a voltage of more than 3 V would need to be applied, based on previous studies of ZnPc.[8] However, tunneling at such high positive voltages became unstable.

In comparison, STML spectra of MPc molecules (M = Zn, Pd, Pt) exhibit the respective Q-band emissions at the same energies as was the case for negative sample bias (Fig. 1a). The STML yield at positive sample bias is at least an order of magnitude smaller, explaining the noisier spectra in Fig. S2. From a previous study focusing on ZnPc, we found indications that there is no plasmonic enhancement of STML at positive bias.[8] The spectra shown here indicate that this may also be the case for the other MPc molecules. In addition to the Q-band emission of the neutral molecule, another faint peak can be observed for all molecules at around 1.35 eV. Previously, we showed that for ZnPc, this can be assigned to the radiative decay of a trion state, i.e., excitonic decay from a transiently charged $ZnPc^-$ molecule.[8] The STML spectra shown in Fig. S2 were acquired with a tip that was not optimized to enhance this spectral feature, which is why we only observed a faint peak at 1.33 eV. While the STML spectrum of PdPc is less clear, PtPc shows a spectral feature at similar energy (ca. 1.36 eV), indicating that this may stem from the radiative decay of the transiently charged $PtPc^-$. Again, the STML spectrum of NiPc is featureless also in this energy range.

3. Temperature-dependent photoluminescence spectra of NiPc

To confirm that NiPc does not show any fluorescence, we performed luminescence spectroscopy of various samples and at different temperatures. Fig. S3 summarizes results for NiPc-(tBu)$_4$ in a fluid 2Me-THF:toluene 1:1 mixture solution at room temperature as well as at

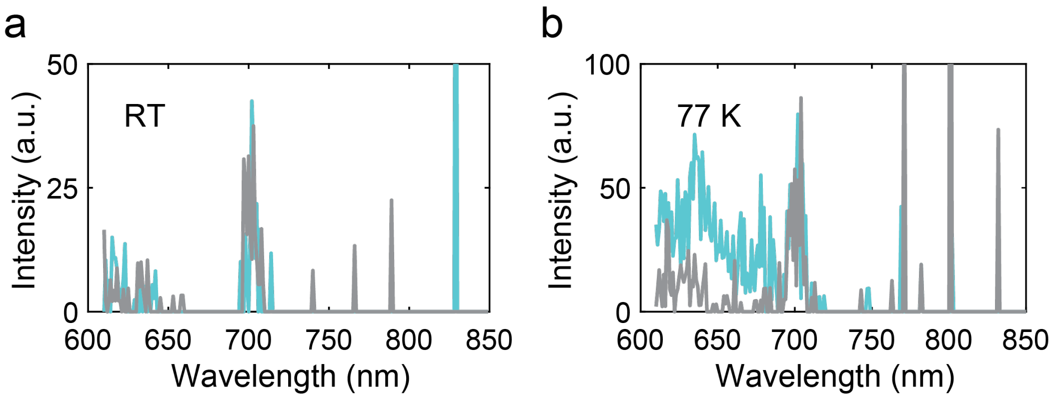

Figure S3. Absence of NiPc-(*t*Bu)$_4$ photoluminescence. (a) Luminescence spectrum of NiPc-(*t*Bu)$_4$ in 2Me-THF:toluene in a 1:1 ratio (grey) taken at room temperature. For comparison, the luminescence spectrum of the pure solvent without NiPc-(*t*Bu)$_4$ is also shown (cyan). (b) Same as (a) but taken at 77 K. For all spectra, an excitation wavelength of 580 nm was used, the detector and excitation bandwidths were set to 4 nm, and no filter was used.

77 K using an excitation wavelength of 580 nm (i.e., a photon energy of 2.138 eV). We used this NiPc derivative to increase solubility, noting that the *t*Bu groups should not have any significant impact on the optical properties (especially unwanted aggregation phenomena). For comparison, also the spectra of the bare solvent are shown. To be able to roughly relate the spectra with those shown in Fig. 2b, we used the same excitation and detector settings as for the measurements of PtPc-(*t*Bu)$_4$, which yielded peak intensities that were more than 2000 times higher than those shown in Fig. S3.

The only spectral feature that can be observed is a very faint peak located at ~700 nm. However, it is also observed in the pure solvent spectra and therefore cannot be assigned to an emission from NiPc-(*t*Bu)$_4$. Based on the absorption spectrum shown in Fig. 2 and considering a possible Stokes shift, we would expect a possible Q-band fluorescence peak of NiPc-(*t*Bu)$_4$ to be located around 670-680 nm with a width of ~20-30 nm. Clearly, there is no such feature visible in any of the spectra. We also carried out optical spectroscopy of NiPc-(*t*Bu)$_4$ (as well as the other MPc derivatives) in a solid state PMMA film, down to a temperature of 6 K. While all other MPc-(*t*Bu)$_4$ samples still show luminescence, NiPc-(*t*Bu)$_4$ still lacks any emission. We note that we repeated measurements using also various smaller excitation wavelengths, down to 350 nm, leading to similar results. To avoid saturation of the detector by scattered light from the excitation source, we did not perform measurements at excitation wavelengths larger than 580 nm.

4. Distance dependence of energy transfer between ZnPc and NiPc

We further investigated the nature of the Q_{NiPc} emission and the underlying energy transfer mechanism by acquiring STML spectra as a function of intermolecular distance R between a ZnPc and a NiPc molecule (Fig. S4). Here, the tip was always positioned at the equivalent location on the ZnPc molecule (marked by a red dot in the inset STM images). We observed NiPc emission only at relatively close distances $R < 2$ nm. At the closest distance $R = 1.45 \pm 0.09$ nm, the RET efficiency[4] was highest with 69±2%, and it quickly decreased with increasing R. For $R \geq 2$ nm, we only observed the fluorescence of ZnPc. We note that the Q_{ZnPc} intensity at $R = 2.94$ nm is larger than at $R = 2.16$ nm. This indicates that there may still be a coupling to the NiPc, but any possible RET is not discernible within the noise. This relatively short-ranged

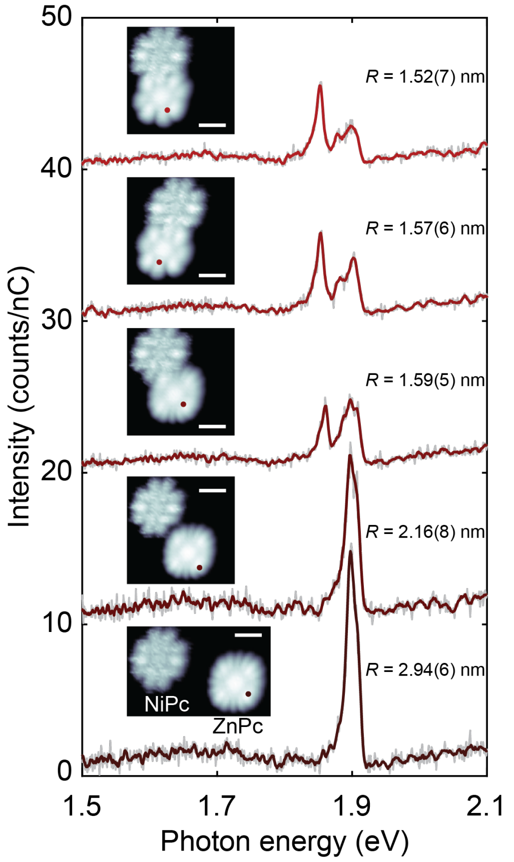

Figure S4. Distance-dependent RET from ZnPc to NiPc. STML spectra taken on a ZnPc molecule with different lateral separation R (center to center) from a neighboring NiPc molecule (see inset STM images; scalebar: 1 nm). Below $R = 2$ nm, fluorescence from ZnPc and NiPc was observed, while for larger separations only ZnPc fluorescence was evident. The STML spectra were acquired in constant-current mode with the tip parked on the ZnPc molecule at the position marked by a red dot in the respective inset topography images, using $V_s = -2.5$ V, $I_t = 200$ pA, $t = 120$ s (for all cases where $R < 2$ nm) as well as $V_s = -2.5$ V, $I_t = 100$ pA, $t = 120$ s (for $R > 2$ nm). The inset STM images were taken in constant-current mode using $V_s = -2.5$ V, $I_t = 10$ pA.

RET is in line with previous observations of RET distance dependence based on STML measurements.[4,9]

5. STS on ZnPc-NiPc dimers

Figure S5 shows a set of dI/dV spectra similar to that shown in Fig. 3c, but for a ZnPc-NiPc dimer. As discussed in the main text, the PIR and NIR positions are identical to those of the monomers, and no continuous transition of the PIR and NIR positions occurs in the interface region between the two molecules. This verifies that the dimers are not electronically hybridized, but the molecules are rather physisorbed next to each other.

6. STML on homodimers

In order to test the nature of intermolecular interactions that lead to the NiPc fluorescence, we investigated NiPc homodimers that were found self-assembled on the surface. As can be seen in Fig. S6a, no molecular emission was observed on this NiPc-NiPc dimer, irrespective of the

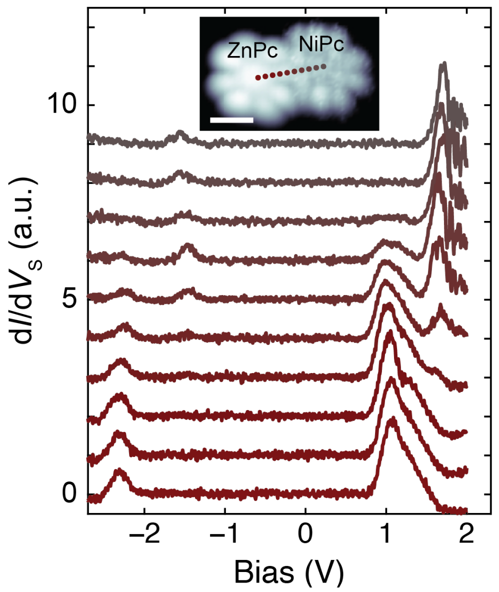

Figure S5. Electronic structure characterization of ZnPc-NiPc dimer. Series of constant-height dI/dV spectra taken along a line across the dimer (see inset), revealing that the individual molecular orbital structures are not altered in the dimer compared to isolated monomers. Feedback loop was opened at the center of ZnPc molecule with $V_s = -2.8$ V, I_t = 50 pA. Inset STM image was acquired at $V_s = -2.5$ V, I_t = 10 pA (scalebar: 1 nm).

tip position. In comparison, a PdPc-PdPc dimer (Fig. S6b) shows strong fluorescence emission. The peak energy varies between 1.80 and 1.82 eV, depending on the tip position. We note that this energy is red-shifted compared to the fluorescence of the monomer, likely due to coherent intermolecular dipole-dipole interaction.[5] We observed similar behavior also for ZnPc-ZnPc dimers.[8] A simple interpretation of homomolecular chains is that excitons are delocalized across the entire chain, which could also be interpreted in a tight-binding picture as excitons hopping between molecules. This again could be considered a special "resonant" case of RET, where donor and acceptor have the same energy. Obviously, such a scenario still cannot induce

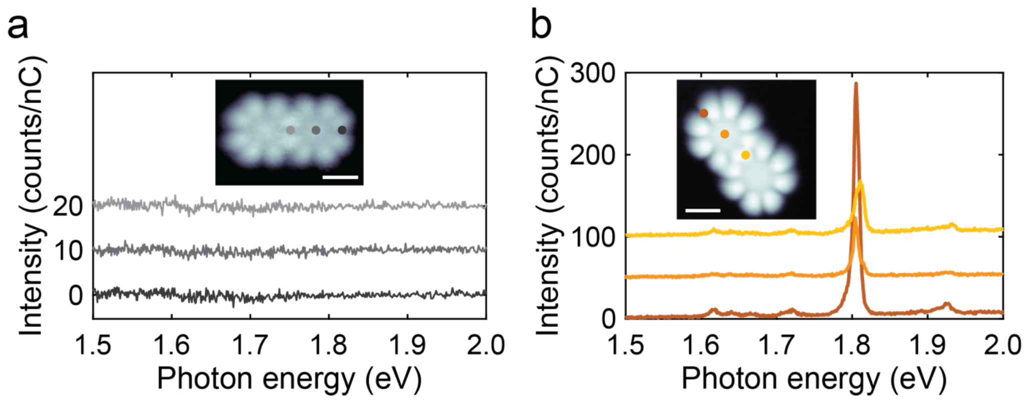

Figure S6. STML on homodimers. (a) STML spectra on a NiPc-NiPc dimer at various positions show no indication of any molecular emission. (b) For comparison, STML spectra on a PdPc-PdPc dimer reveal a strong molecular emission at about 1.8 eV, as well as some vibrational side peaks. Inset STM images were acquired at I_t = 10 pA, $V_s = -2.6$ V (scalebar: 1 nm). STML acquisition parameters: $V_s = -2.6$ V, I_t = 10 pA, t = 120 s.

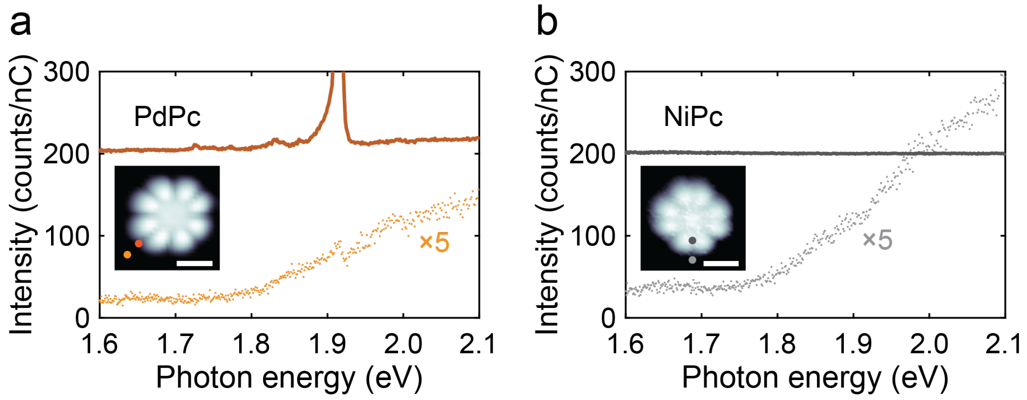

Figure S7. Plasmon-induced STML. (a) STML spectrum on (top) and off (bottom) PdPc. In the latter case, a Fano resonance is observed at 1.92 eV when the tip is located close (< 2 nm) to the PdPc but no direct tunneling into the molecules occurs. (b) STML spectrum on (top) and off (bottom) NiPc. For the latter, no Fano resonance is observed, and all observed STML intensity is identical to that of spectra taken far away from any molecule. Inset STM images were acquired at I_t = 10 pA, V_s = −2.6 V (scalebar: 1 nm). STML acquisition parameters: I_t = 100 pA, V_s = −2.6 V, t = 120 s.

fluorescence in NiPc, presumably because the ISC in the initially excited NiPc occurs much faster than the energy transfer of a (π,π^*) exciton to a neighboring NiPc.

7. Spatial dependence of STML

In order to test whether molecular fluorescence of NiPc can be activated by plasmon-induced excitation, we compared STML spectra taken on top of the molecule (i.e., while still directly tunneling through it) with those taken when the tip was positioned laterally away from the molecule to prevent direct tunneling. For comparison, we first show results for a PdPc molecule (Fig. S7a). When the tip is positioned at the edge of the macrocycle (i.e. resonant tunneling through the molecule is still possible), the STML spectrum (red) shows an intense peak at 1.91-1.92 eV. We note a slight blueshift compared to the spectrum in Fig. 1 may be due to a reduced Lamb shift.[10] When placing the tip off the molecule (see points marked in the inset STM image), the STML spectrum is dominated by the plasmon resonance of the tip-sample nanocavity (often referred to as nanocavity plasmons (NCPs)), but a faint Fano resonance is visible at 1.92 eV, which is the hallmark of coherent plasmon-exciton coupling and hence leads to faint molecular fluorescence.[11]

In comparison, NiPc STML spectra using the same tip show no indication of molecular fluorescence, irrespective of where the tip is located during the STML acquisition (Fig. S7b). We note that the faint broad feature between 1.8 eV and 1.9 eV cannot be ascribed to the NiPc, as it was also visible in NCP spectra on NaCl/Ag(111) further away from the molecule, using the same tip. We verified that even at applied voltages of $V_s = \pm 3$ V, the results remain the same. We therefore conclude that plasmon-induced excitation is not able to activate NiPc fluorescence, for the voltages used. We note that the voltage magnitudes used for the NCP excitation were much larger than $E(Q_{NiPc})$. The observations could be explained by assuming that also higher-energy NCPs can transfer energy to the NiPc and excite it into a vibrationally hot S_1 state, leading to ISC.

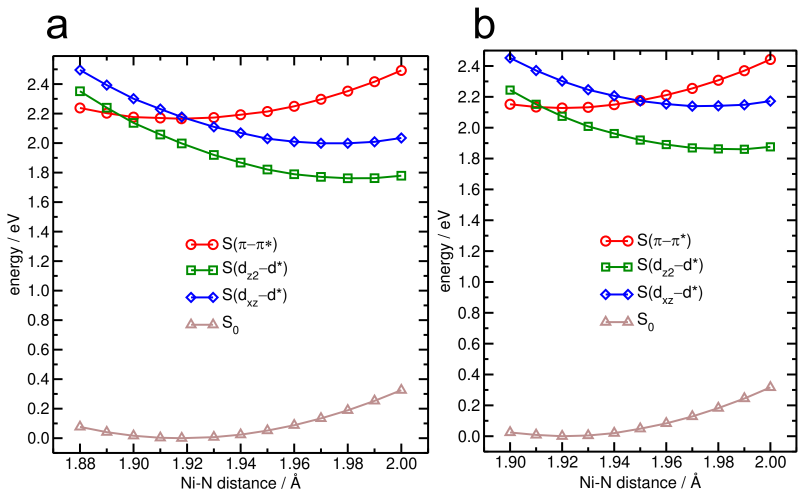

Figure S8. TDDFT excited-state energy profiles of NiPc for varying Ni-N distances. (a) Using the PBE0 hybrid functional; (b) using the range-separated hybrid functional CAM-B3LYP. Here, only singlet excited states were considered. For comparison, the optimized Ni-N distance in the ground state was found at 1.918 Å (PBE0) and 1.920 Å (CAM-B3LYP), respectively.

8. Theoretical details and analysis

All density functional theory (DFT) calculations were performed using the quantum chemistry package Gaussian 09 Rev. D.01[12] with the PBE0[13] and CAM-B3LYP[14] exchange-correlation functionals and the SDD basis set, which applies an effective core potential for the Pt and Pd atoms[15] and the D95 basis set for H, C, N, F and O atoms.[16] Excited state energy profiles were calculated using time-dependent DFT (TDDFT) linear response theory along the Ni-N reaction coordinate at geometries optimized with all four Ni-N bonds constrained to the same distance. Due to occurring triplet instabilities, additional TDDFT calculations were carried out in the Tamm-Dancoff approximation to obtain more reliable energies of the triplet states relative to the singlet manifold. Molecular orbitals (MOs) were visualized using Avogadro 1.2.0.[17]

From the results of TDDFT, we found that there is a number of *d-d* exited states located at energies below that of the π-π* singlet state (see also Table S1). The molecular structures optimized in these two types of electronic states mostly differ in the bond length between the central Ni atom and the four neighboring isoindole N_p atoms, hence we can identify the Ni-N bond distance as a suitable reaction coordinate. A corresponding vibrational mode that changes this bond distance in our calculations is the symmetric Ni-N stretch vibration at 1411 cm^{-1} ≈ 175 meV. To explore the reasons why fluorescence only occurs upon resonant excitation of the lowest excited π-π* singlet state, we scanned the potential energies of the closest-lying excited states along the Ni-N coordinate. Figure S8a shows the corresponding potential curves obtained from TDDFT calculations with the PBE0 hybrid functional. Due to the occurring triplet instabilities only the singlet manifold is considered here. Two crossings of the π-π* singlet state (red) with other singlet excited states – namely the d_{z^2}-$d^*_{x^2-y^2}$ state (green) as well as the degenerate d_{xz}-$d^*_{x^2-y^2}$ and d_{yz}-$d^*_{x^2-y^2}$ states (blue) – can be seen very close to the minimum of the π-π* state. The π-π* / d_{xz}-$d^*_{x^2-y^2}$ crossing is located only about 1 meV above the π-π* minimum, whereas the π-π* / d_{z^2}-$d^*_{x^2-y^2}$ intersection lies about 0.02 eV higher. Thus, for non-resonant excitation both crossings should be accessible, leading to a depopulation of the bright

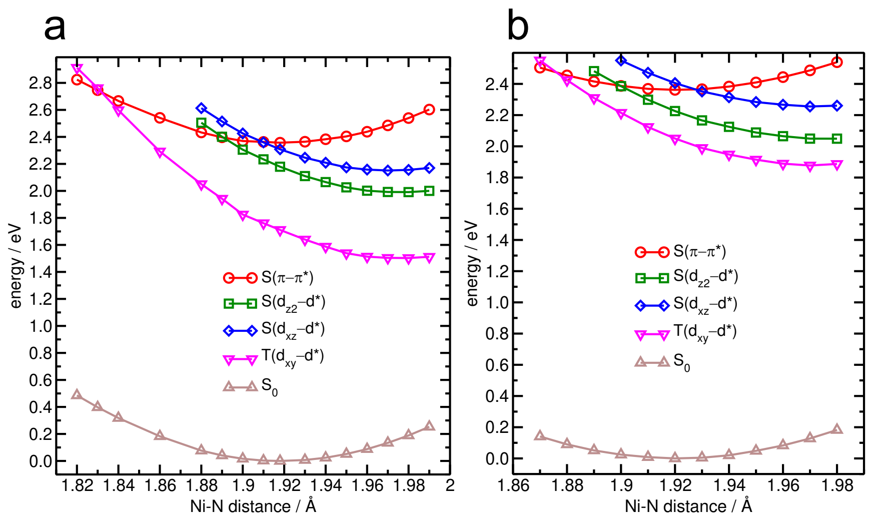

Figure S9. TDDFT excited-state energy profiles of NiPc for varying Ni-N distances. Here, we used the Tamm-Dancoff approximation to better assess triplet states. (a) Using the PBE0 hybrid functional; (b) using the range-separated hybrid functional CAM-B3LYP.

π-π* state into the dark d-d* states. We note that for the S0 ground state we found the optimized Ni-N distance at 1.918 Å (PBE0) and 1.920 Å (CAM-B3LYP), respectively. Hence, S_0 and S_1 are nested states, as usually found for phthalocyanines.

The exact positions of the theoretically predicted crossing points however depend on a number of factors such as the choice of exchange-correlation functional, basis set, and environment model (i.e. NaCl substrate or solvent). While we will comment on the latter below, to gauge the effect of the exchange-correlation functional, we recalculated the potential energy curves using the range-separated hybrid functional CAM-B3LYP (see Fig. S8b). We can see that the π-π* / d_{xz}-$d^*_{x^2-y^2}$ crossing is now slightly less accessible at 0.05 eV above the π-π* minimum, while the π-π* / d_{z^2}-$d^*_{x^2-y^2}$ intersection now lies only 5 meV above the π-π* minimum. From the comparison, it is already obvious that we cannot identify into which d-d* state exactly the ISC from the π-π* state occurs.

Invoking the Tamm-Dancoff approximation allows us to assess more reliably the relative energies of singlet and triplet states. Table S1 summarizes the first 17 excitations found for NiPc and which MOs are involved (see also Table S2 for orbital identification), using the PBE0 functional. Calculations as a function of Ni-N distance reveal a crossing between the π-π* singlet state and the d_{xy}-$d^*_{x^2-y^2}$ triplet state at 0.37 eV above the π-π* minimum (Fig. S9a). Using the CAM-B3LYP functional, on the other hand, the energy difference is only 0.11 eV (Fig. S9b). This opens up the possibility for intersystem crossing followed by radiationless relaxation back to the ground state via a number of deep-lying triplet states (Table S1) in the case of non-resonant excitation.

Concerning the effect of the environment, we assume that the flat adsorption geometry of NiPc on NaCl will likely suppress out-of-plane vibrations and also induce a friction on the Ni-N breathing mode. In addition, the interaction of NiPc with the Na^+ and Cl^- ions will have an effect on its electronic structure and thus further change the intersection points with respect to the π-π* minimum. Besides, we note that absolute excited state energies from TDDFT generally differ from experimental values by typically 0.2-0.4 eV.[18,19] We therefore conclude that our TDDFT calculations neither permit to identify which particular d-d* state is responsible for the fast ISC from the π-π* state, nor can we quantify the ISC activation barrier in the lab setup with

sufficient accuracy. Nevertheless, the TDDFT results give us a qualitative picture, rationalizing that there actually is an activation barrier for ISC due to an intersection of the π-π^* singlet state with a d-d* state, as well as identifying that the "reaction coordinate" is the Ni-N bond length. We note that also the energy of the symmetric Ni-N stretch vibration at ca. 175 meV gives an indication of the minimum energy required to enable ISC from the vibrational ground state of the π-π^* singlet state.

9. NiPc vs. HPc$^-$

At first glance, there seem to be circumstantial similarities in the experimental observation of NiPc and those of deprotonated free-base phthalocyanine, i.e. HPc$^-$.[20] Therefore, we would first like to comment on a potential unwanted contamination of our sample with free-base H_2Pc. First of all, no experiments with H_2Pc have ever been performed in the UHV system used here. NiPc itself is a very stable compound, and does not dissociate by itself or in the course of sublimation (at temperatures below 350°C).[21] In order to reduce any possible H_2Pc contamination of our source material, we utilized the fact that its vapor pressure is about an order of magnitude larger than that of NiPc. [22] We therefore repeatedly degassed the NiPc source material in UHV by running the Knudsen cell evaporator for up to one hour at the target deposition temperature (300-350°C). We confirmed the overall absence of H_2Pc by taking a sample of the degassed material out of the crucible and performing optical spectroscopy in solution (see Section 1). From the absence of any fluorescence, we can estimate that a potential H_2Pc contamination of the degassed NiPc source material would be less than 1%.

Our STM results confirm the purity of the degassed NiPc source upon cold deposition onto the NaCl/Ag(111) substrate. If there would have been H_2Pc present, this would have become obvious in STM images at positive bias, as the H_2Pc LUMO is not degenerate, leading to a twofold-symmetric feature in STM.[23] Furthermore, H_2Pc does not deprotonate spontaneously but requires large tunneling voltages $V_s > 3.2$ V, and HPc$^-$ would not be the final product, but deprotonation can continue to form Pc^{2-} as well, which again has different structural, electronic and electronic and optical properties.[20] In contrast to all this, we only found one species in all our STM images, with reproducibly identical properties in STS and STML measurements, as reported here.

Finally, we would like to emphasize various differences in the observables of NiPc in our study vs. HPc$^-$ reported by Vasilev *et al.*[20] The onset energies of the NiPc PIR and NIR are slightly different, leading to a gap that is 0.2 eV larger than that of HPc$^-$. STM images and dI/dV maps of the LUMO (see Fig. 1b) exhibit a fourfold symmetry, whereas HPc$^-$ shows a reduced symmetry. The noise recorded in time traces of the tip-sample distance above NiPc is of a broadband nature, whereas that of HPc$^-$ exhibits a four-state discrete telegraph noise due to switching of the remaing central H atom between one of the four possible isoindole N_p atoms. Moreover, NiPc shows a noisy appearance in STM imaging for all voltages, whereas the HPc$^-$ telegraph noise only occurred at negative voltages.

In summary, we can exclude the possibility of accidentally having measured HPc$^-$, and we have confirmed the purity of our NiPc source material.

Table S1. TDDFT/PBE0 eigenvalues (for all excited states below 3 eV), eigenvectors, and oscillator strengths f using the Tamm-Dancoff approximation for the ground-state optimized NiPc structure. Only molecular orbital contributions with a weight of more than 10% are listed. MO 141 and 142 are the HOMO and LUMO, respectively. The π-π^* singlet state corresponds to #10 and #11, owing to the degenerate LUMO and LUMO+1.

#	Spin	Energy (eV)	f	MO contributions and weights
1	triplet	0.4679	0.0000	132 → 144 (98.4%)
2	triplet	0.7796	0.0000	126 → 144 (25.4%)<br>127 → 144 (22.1%)<br>139 → 144 (24.8%)<br>140 → 144 (18.2%)
3	triplet	0.7796	0.0000	126 → 144 (22.1%)<br>127 → 144 (25.4%)<br>139 → 144 (18.2%)<br>140 → 144 (24.8%)
4	triplet	1.3488	0.0000	141 → 142 (87.9%)<br>141 → 143 (10.2%)
5	triplet	1.3488	0.0000	141 → 142 (10.2%)<br>141 → 143 (87.9%)
6	triplet	1.7110	0.0000	124 → 144 (82.8%)<br>134 → 144 (15.6%)
7	singlet	2.1790	0.0000	132 → 144 (95.6%)
8	singlet	2.3080	0.0000	126 → 144 (37.5%)<br>139 → 144 (47.5%)
9	singlet	2.3080	0.0000	127 → 144 (37.5%)<br>140 → 144 (47.5%)
10	singlet	2.3571	0.5797	141 → 142 (57.7%)<br>141 → 143 (27.1%)
11	singlet	2.3571	0.5797	141 → 142 (27.1%)<br>141 → 143 (57.7%)
12	singlet	2.7192	0.0000	124 → 144 (78.1%)<br>134 → 144 (20.3%)
13	triplet	2.8144	0.0000	139 → 142 (32.7%)<br>139 → 143 (10.7%)<br>140 → 142 (10.7%)<br>140 → 143 (32.7%)
14	triplet	2.8683	0.0000	139 → 142 (42.0%)<br>140 → 143 (42.0%)
15	triplet	2.8921	0.0000	139 → 142 (42.4%)<br>140 → 143 (42.4%)
16	triplet	2.9165	0.0000	141 → 144 (99.4%)
17	singlet	2.9309	0.0000	141 → 144 (99.5%)

Table S2. Molecular orbital isosurface plots (isovalue = 0.02) calculated for the optimized ground state geometry using the PBE0 functional. The selected orbitals have the highest contributions to the excited states plotted S7.

LUMO+2 (MO 144)	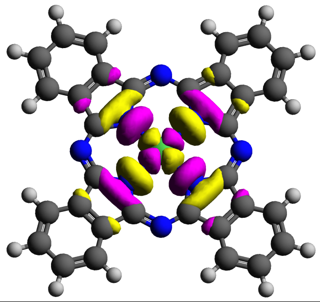	HOMO-2 (MO 139)	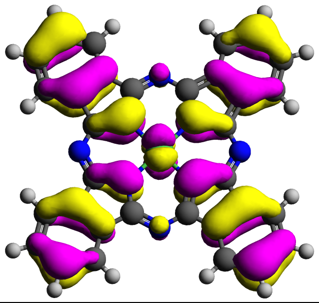
LUMO+1 (MO 143)	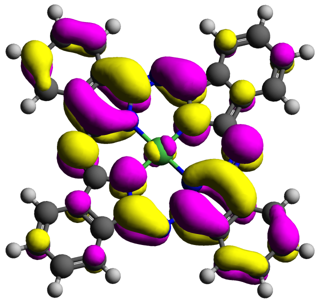	HOMO-9 (MO 132)	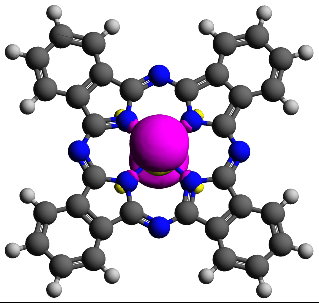
LUMO (MO 142)	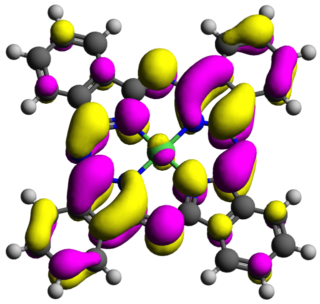	HOMO-14 (MO 127)	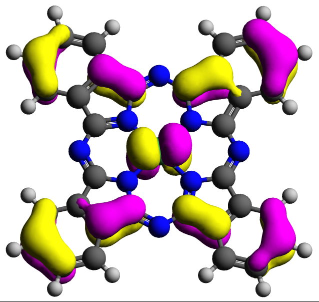
HOMO (MO 141)	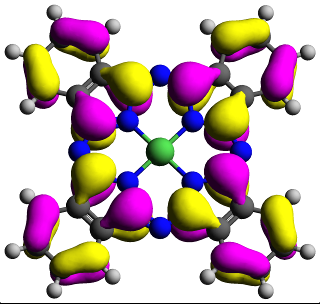	HOMO-15 (MO 126)	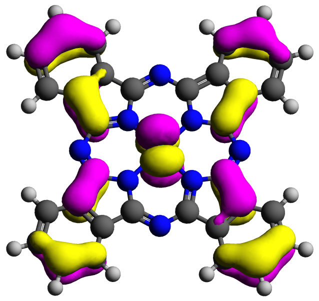
HOMO-1 (MO 140)	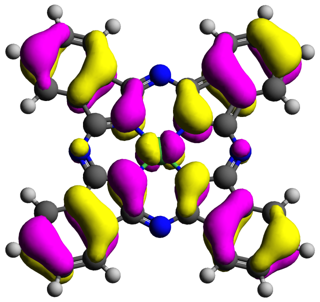	HOMO-17 (MO 124)	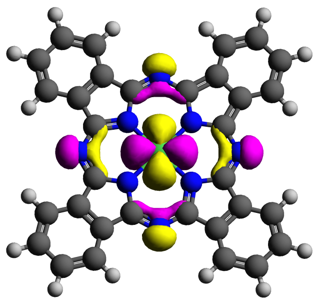

References

1 Hung, T.-C., Kiraly, B., Strik, J. H., Khajetoorians, A. A. & Wegner, D., Plasmon-Driven Motion of an Individual Molecule, *Nano Lett.* **21**, 5006 (2021).

2 Zhang, C., Gao, B., Chen, L. G., Meng, Q. S., Yang, H., Zhang, R., Tao, X., Gao, H. Y., Liao, Y. & Dong, Z. C., Fabrication of silver tips for scanning tunneling microscope induced luminescence, *Rev. Sci. Instrum.* **82**, 083101 (2011).

3 Iwami, M., Uehara, Y. & Ushioda, S., Preparation of silver tips for scanning tunneling microscopy imaging, *Rev. Sci. Instrum.* **69**, 4010 (1998).

4 Cao, S., Rosławska, A., Doppagne, B., Romeo, M., Féron, M., Chérioux, F., Bulou, H., Scheurer, F. & Schull, G., Energy funnelling within multichromophore architectures monitored with subnanometre resolution, *Nat. Chem.* **13**, 766 (2021).

5 Zhang, Y., Luo, Y., Zhang, Y., Yu, Y.-J., Kuang, Y.-M., Zhang, L., Meng, Q.-S., Luo, Y., Yang, J.-L., Dong, Z.-C. & Hou, J. G., Visualizing coherent intermolecular dipole–dipole coupling in real space, *Nature* **531**, 623 (2016).

6 Liao, M. S. & Scheiner, S., Electronic structure and bonding in metal phthalocyanines, Metal=Fe, Co, Ni, Cu, Zn, Mg, *J. Chem. Phys.* **114**, 9780 (2001).

7 Uhlmann, C., PhD thesis (2013).

8 Hung, T.-C., Robles, R., Kiraly, B., Strik, J. H., Rutten, B. A., Khajetoorians, A. A., Lorente, N. & Wegner, D., Bipolar single-molecule electroluminescence and electrofluorochromism, *Phys. Rev. Res.* **5**, 033027 (2023).

9 Kong, F.-F., Tian, X.-J., Zhang, Y., Zhang, Y., Chen, G., Yu, Y.-J., Jing, S.-H., Gao, H.-Y., Luo, Y., Yang, J.-L., Dong, Z.-C. & Hou, J. G., Wavelike electronic energy transfer in donor–acceptor molecular systems through quantum coherence, *Nat. Nanotechnol.* **17**, 729 (2022).

10 Zhang, Y., Meng, Q. S., Zhang, L., Luo, Y., Yu, Y. J., Yang, B., Zhang, Y., Esteban, R., Aizpurua, J., Luo, Y., Yang, J. L., Dong, Z. C. & Hou, J. G., Sub-nanometre control of the coherent interaction between a single molecule and a plasmonic nanocavity, *Nat. Commun.* **8**, 15225 (2017).

11 Kröger, J., Doppagne, B., Scheurer, F. & Schull, G., Fano Description of Single-Hydrocarbon Fluorescence Excited by a Scanning Tunneling Microscope, *Nano Lett.* **18**, 3407 (2018).

12 Gaussian 09 Revision D.01 (Gaussian Inc., Wallingford, CT, 2009).

13 Adamo, C. & Barone, V., Toward reliable density functional methods without adjustable parameters: The PBE0 model, *J. Chem. Phys.* **110**, 6158 (1999).

14 Yanai, T., Tew, D. P. & Handy, N. C., A new hybrid exchange-correlation functional using the Coulomb-attenuating method (CAM-B3LYP), *Chem. Phys. Lett.* **393**, 51 (2004).

15 Andrae, D., Häussermann, U., Dolg, M., Stoll, H. & Preuss, H., Energy-adjusted ab initio pseudopotentials for the second and third row transition elements, *Theor. Chim. Acta* **77**, 123 (1990).

16 Dunning, T. H. & Hay, P. J., Gaussian Basis Sets for Molecular Calculations. In *Methods of Electronic Structure Theory* (ed. Henry F. Schaefer) 1-27 (Springer US, 1977).

17 Hanwell, M. D., Curtis, D. E., Lonie, D. C., Vandermeersch, T., Zurek, E. & Hutchison, G. R., Avogadro: an advanced semantic chemical editor, visualization, and analysis platform, *J. Cheminformatics* **4**, 17 (2012).

18 Laurent, A. D. & Jacquemin, D., TD-DFT benchmarks: A review, *Int. J. Quantum Chem.* **113**, 2019 (2013).

19 Liang, J. S., Feng, X. T., Hait, D. & Head-Gordon, M., Revisiting the Performance of Time-Dependent Density Functional Theory for Electronic Excitations: Assessment of 43 Popular and Recently Developed Functionals from Rungs One to Four, *J. Chem. Theory Comput.* **18**, 3460 (2022).
20 Vasilev, K., Doppagne, B., Neuman, T., Roslawska, A., Bulou, H., Boeglin, A., Scheurer, F. & Schull, G., Internal Stark effect of single-molecule fluorescence, *Nat. Commun.* **13**, 677 (2022).
21 Yase, K., Takahashi, Y., Arakato, N. & Kawazu, A., Evaporation Rate and Saturated Vapor-Pressure of Functional Organic Materials, *Jpn. J. Appl. Phys.* **34**, 636 (1995).
22 Basova, T., Semyannikov, P., Plyashkevich, V., Hassan, A. & Igumenov, I., Volatile Phthalocyanines: Vapor Pressure and Thermodynamics, *Crit. Rev. Solid State* **34**, 180 (2009).
23 Doppagne, B., Neuman, T., Soria-Martinez, R., Lopez, L. E. P., Bulou, H., Romeo, M., Berciaud, S., Scheurer, F., Aizpurua, J. & Schull, G., Single-molecule tautomerization tracking through space- and time-resolved fluorescence spectroscopy, *Nat. Nanotechnol.* **15**, 207 (2020).
